# All or nothing: Switch to high current reproductive investment under risk of starvation in male kelp crab

**DOI:** 10.1002/ece3.6131

**Published:** 2020-03-07

**Authors:** Katrin Pretterebner, Luis M. Pardo

**Affiliations:** ^1^ Programa de Doctorado en Biología Marina Facultad de Ciencias Universidad Austral de Chile Valdivia Chile; ^2^ Laboratorio Costero de Calfuco Facultad de Ciencias Instituto de Ciencias Marinas y Limnológicas Universidad Austral de Chile Valdivia Chile; ^3^ Centro de Investigación de Dinámica de Ecosistemas Marinos de Altas Latitudes (IDEAL) Valdivia Chile

**Keywords:** crustacean, food availability, reproduction, temperature, terminal investment, vasa deferentia

## Abstract

One of the key features in reproduction of polygynous species is seminal recovery after mating. However, it is poorly known how environmental factors affect the recuperation period of seminal material. This study aims to test plasticity in recovery of seminal reserves in response to distinct environmental conditions of the kelp crab *Taliepus dentatus*. Male crabs were maintained after depletion of seminal reserves in one of eight different treatments in a factorial design of temperature (12 and 16°C), food availability (with alimentation and food deprivation), and time period (15 and 30 days), simulating different environmental situations in the laboratory to which the crab might be exposed to along its distribution. Temperature and food availability modulated the seminal recovery period in *T. dentatus*. Complete replenishment was reached within 30 days in all treatments (i.e., 12 and 16°C each with alimentation and food deprivation), but the highest recovery index was found in crabs without food provision (16°C). In this condition, the recovery index was twice as high compared with males maintained at a similar temperature but with feeding. Prolonged starvation at 16°C may be extremely stressful conditions for male crabs, during which risk to die probably triggered a concentration of the reproductive effort in favor of immediate reproduction. This suggests that plasticity of energy allocation toward reproduction may be expressed during extremely suboptimal conditions, which might be a similar strategy as proposed by the terminal investment hypothesis. The generally relatively fast seminal recovery regardless of the temperature may explain the kelp crab's continuous mating throughout the year.

## Graphical abstract

In this study, we demonstrate plasticity in recovery of seminal reserves in response to distinct environmental conditions in the kelp crab *Taliepus dentatus*. We show that the recovery index at 16°C within 30 days was twice as high in starved compared with fed crabs. Prolonged starvation at 16°C may be extremely stressful conditions for male crabs, during which risk to die probably triggered a concentration of the reproductive effort in favor of immediate reproduction. This suggests that plasticity of energy allocation toward reproduction may be expressed during extremely suboptimal conditions, which might reflect a similar strategy as proposed by the terminal investment hypothesis.

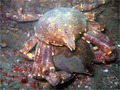

## 1 INTRODUCTION

Environmental factors, such as temperature, photoperiod, or availability of food, are known to influence a variety of reproductive attributes of invertebrates in general and crustacean species in particular. These include life history traits such as size at maturity, number of broods, fecundity and size of eggs, as well as key reproductive processes like ovarian development and synthesis of vitellogenin (Brante, Fernández, Eckerle, Mark, & Pörtner, 2003; Burmeister & Sainte‐Marie, 2010; Colpo & López Greco, 2017; Fischer & Thatje, 2008; Fischer, Thatje, & Brey, 2009; Thatje & Hall, 2016; Thongda, Chung, Tsutsui, Zmora, & Katenta, 2015). In these studies, environmental variability has been associated with seasonal and latitudinal fluctuations. To date, most studies in this research field are biased toward females, which are supposed to have a greater energetic investment in reproduction than males. Little is known how environmental factors influence male reproductive traits (e.g., size at maturity; Burmeister & Sainte‐Marie, 2010). In general, the traditional assumption of limitless and cheap sperm of males (Levitan & Petersen, 1995) has been changed by the recognition that sperm are accompanied by accessory fluids and/or spermatophores, which may be costly to produce (Dewsbury, 1982). Further indication that the production of seminal material (i.e., both sperm and seminal fluids) incurs energetic costs is supported by the presence of ejaculate allocation strategies as well as prudent ejaculate expenditure described in terrestrial insects, birds, fishes, and crustaceans (Wedell, Gage, & Parker, 2002).

Recovery period of seminal reserves after mating may be a key feature in reproduction as it influences the available seminal material stock and as a consequence the male's mating capacity (Sainte‐Marie, 2007). So far, sperm or seminal regeneration rates have been determined in various decapod species (Bugnot & López Greco, 2009; Kendall, Wolcott, Wolcott, & Hines, 2001; Pascual et al., 1998; Pretterebner, Pardo, & Paschke, 2019; Sato, Ashidate, Jinbo, & Goshima, 2006; Sato & Goshima, 2006). However, the key environmental variable temperature, which affects the rate of the energetic metabolism in ectotherms, was used only in few of these studies as a factor. Examples with a temperature‐dependent production of seminal material/sperm are the shrimp *Penaeus setiferus* (Pascual et al., 1998) and the southern king crab *Lithodes santolla* (Pretterebner et al., 2019). On one hand, in the tropical shrimp *P. setiferus,* spermatophores are replaced faster at 33°C (6 days) compared with 25°C (8 days) (Pascual et al., 1998). On the other hand, in the temperate/cold‐temperate species *L. santolla,* full seminal recovery requires more than 30 days and is twice as fast at 9°C than 12°C (Pretterebner et al., 2019).

Another important environmental factor is the availability of food resources, which can constrain organisms in their allocation of energy to distinct requirements (i.e., maintenance of body functions, somatic growth, and reproduction). During food limitation, energy allocation to maintain the basal maintenance functions (e.g., ion and acid–base regulation, ventilation, and circulation) is typically prioritized in aquatic invertebrates (Sokolova, Frederich, Bagwea, Lannig, & Sukhotin, 2012), and growth, reproduction, or activity is reduced (Comoglio, Smolko, & Amin, 2005; Terwilliger & Dumler, 2001; Wen, Chen, Ku, & Zhou, 2006). Thus, variability in food availability is likely to lead to condition‐dependent investment in reproduction (Boggs, 1992). Evidence that production of ejaculate or sperm is dependent on availability of food comes from studies with different taxa such as the common bedbug *Cimex lectularius* (Kaldun & Otti, 2016), the mosquitofish *Gambusia holbrooki* (O’Dea, Jennions, & Head, 2014), and lizards (Kahrl & Cox, 2015). For example, in the common bedbug, food positively affects ejaculate production and males that receive large meals produce significantly more sperm and seminal fluid compared with food‐restricted ones (Kaldun & Otti, 2016). Results of the mosquitofish and lizards outlined that the rate of sperm production was associated with the amount of food ingested.

Evidence of the combined effect of temperature and food availability on reproductive aspects exists for ectothermic fish species, such as the Atlantic cod *Gadus morhua* (Yoneda & Wright, 2005) and the coral reef fish *Acanthochromis polyacanthus* (Donelson, Munday, McCormick, Pankhurst, & Pankhurst, 2010). For example, in males of the Atlantic cod, testicular weight and the gonadosomatic index are larger at higher local temperature with a high ration of food suggesting a higher sperm production (Yoneda & Wright, 2005). When environmental factors act in a combined form, the effects of food availability can be modified by temperature. For example, in the coral reef fish, elevated temperatures in the context of climate change reduce sperm production, and availability of food modulates this response (Donelson et al., 2010). At 28 and 30°C with low food, males have more spermatozoa compared with those in high food regimes. In contrast, at 31.5°C, males with a low food ration have fewer spermatozoa than those with a high ration. The combined effect of temperature and food availability on the recovery rate of seminal reserves after their depletion has not been examined so far in any crab species.

**Figure 1:**
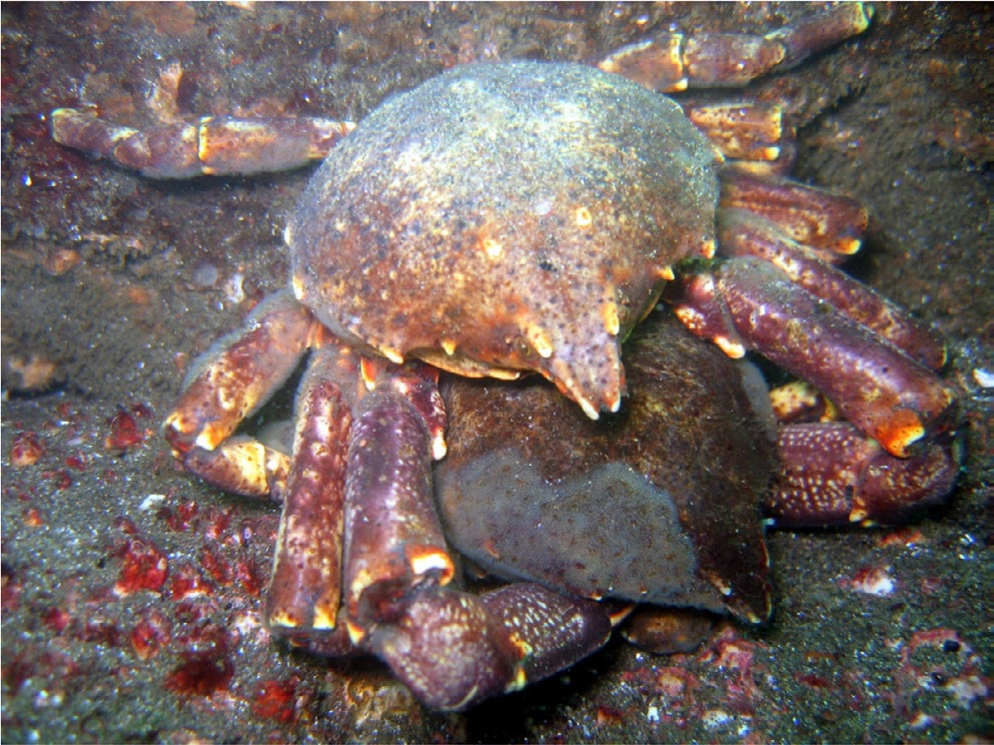
Individuals of *Taliepus dentatus* during copulatory guarding. Photograph used with permission of Luis M. Pardo

The kelp crab *Taliepus dentatus* (H. Milne Edwards, 1834; Figure 1) is a subtidal majoid species with an extended distribution along the Pacific coast from Peru (12°S) to the Chilean Patagonia (51°S) (Fagetti & Campodonico, 1971). This species inhabits preferentially kelp forests formed by several species of brown algae. It supports a small artisanal fishery which is concentrated in the austral regions of Chile (Chilean 5‐year mean annual landing around 19.4 tons, annual statistical report from Servicio Nacional de Pesca, 2013–2017). Capture of this species is regulated by the prohibition of harvesting females. Oviposition in *T. dentatus* has been registered year‐round (Fagetti & Campodonico, 1971; Retamal et al., 2009). Studies on *T. dentatus* have been mainly focused on thermal tolerance of larvae (Carreja, Fernández, & Agusti, 2016; Storch, Fernández, Navarrete, & Pörtner, 2011; Storch et al., 2009) and maternal investment of females (Baldanzi, Storch, Navarrete, Graeve, & Fernández, 2018). Knowledge on the male reproductive biology is absent despite their local socioeconomic relevance. This study aims to test the combined effect of temperature and food availability on male seminal recovery of a kelp crab, simulating different environmental scenarios in the laboratory to which the crab might be exposed to along its distribution.

## 2 MATERIAL AND METHODS

Similarly sized physiologically mature males of *T. dentatus* were collected by scuba diving at the end of October 2017 from Los Molinos Bay (39°51′16.7″S; 73°23′40.3″W) in southern Chile. Crabs were transported to the Laboratorio Costero de Calfuco of the Universidad Austral de Chile where they were maintained in 500 L tanks with flowing seawater, air supply, and ad libitum food until the start of the experiment. Prior to the beginning of the experiment, crabs were maintained 15 days at a temperature similar to that present in natural conditions due to flowing seawater. As samples were collected during the austral spring (end of October), during which the mean sea surface temperature likely increases to approximately 14°C (Garcés‐Vargas, Ruiz, Pardo, Nuñez, & Pérez‐Santos, 2013; data only available for September 11°C and January 15°C), the thermal shock was minimal when they were placed in the experimental conditions. Moreover, this species lives in zones of coastal upwelling, where great changes in temperature can occur during short periods due to the cycle of coastal upwelling and its relaxation (Pardo, Mora‐Vásquez, & Garcés‐Vargas, 2012, Figure 4). All experimental conditions were started simultaneously in mid‐November 2017.

Crabs were stimulated for electroejaculation on various successive days until no seminal material was obtained, to ensure standardization of empty vasa deferentia. Electroejaculation was performed through short electric shocks (12 V AC; 10 s), which were repeated 10 times with brief breaks of 5 s (Pretterebner et al., 2019; Soundarapandian, 2013). During electroejaculation, two electrodes were placed ventrally at the base between the third and fourth pair of pereiopods of crabs. Then, crabs were randomly assigned to one of eight different treatments in a fully factorial design including temperature (12 and 16°C), food availability (with alimentation and food deprivation), and time period (15 and 30 days). Crabs were maintained in individual containers of ~18 L with flowing seawater and air supply. The lower experimental temperature was maintained by placing the individual containers in a water bath with chillers (11.9 ± 0.05°C, all values are means ± standard errors). The higher experimental temperature (15.9 ± 0.75°C) was previously adjusted in a large tank by a submersible aquarium heater with a digital controller and from there it was distributed to the individual containers with crabs. This temperature condition was conducted in an acclimatized room to maintain the stable temperature. These two experimental temperatures are referred to as 12 and 16°C in the text, reflecting the mean surface temperatures in the estuary of Valdivia and the adjacent coast between the austral autumn and spring (around 12°C) and during the austral summer (15.3°C, fluctuations do not exceed 17.3°C), respectively (Garcés‐Vargas et al., 2013). Temperature was measured daily. Individuals in the ad libitum treatment were fed once per day ad libitum with mussels. All individual containers were siphoned daily to remove feces and possible food leftovers. We sacrificed crabs after three time periods: 0, 15, and 30 days.

After the corresponding recovery periods, we sacrificed crabs (anesthetized by thermal shock: −20°C for 20 min), and both vasa deferentia (i.e., structure of storage of seminal material) and the hepatopancreas were extracted. The dry weight of vasa deferentia was determined (oven‐dried for 4 days at 70°C and weighed to a precision of 0.0001 g; *n* = 5 in each group, apart from *n* = 4 in control, initial crabs with electroejaculation and crabs after 30 days at 16°C with and without food) to subsequently estimate seminal recovery by means of the calculation of the recovery index (see below).

In crustaceans, the hepatopancreas is an organ where digestive processes occur and is the main site of energy storage from which resources are mobilized during molting (Sánchez‐Paz, García‐Carreño, Hernández‐López, Muhlia‐Almazán, & Yepiz‐Plascencia, 2007). The weight of the hepatopancreas has been suggested as an indicator of the body condition (Sagi & Raánan, 1988). The dry weight of the hepatopancreas and complete body was determined (oven‐dried for 4 days at 70°C and weighed to a precision of 0.01 g; *n* = 5 in each group, apart from *n* = 4 in crabs after 30 days at 16°C with and without food). To control for the body weight of the crabs, the hepatosomatic index (expressed as a percentage) was calculated as the ratio between hepatopancreas and total body dry weight multiplied by 100.

### 2.1 Data analyses and statistics

The recovery index (RI; Pretterebner et al., 2019) was calculated to estimate seminal recuperation for each treatment. RI refers to the recovery rate expressed as the percentage change in dry weight of vasa deferentia between initially electroejaculating individuals and after the corresponding experimental period in relation to dry weight of the vasa deferentia of individuals used as control (i.e., without electroejaculation). Dry weight of the vasa deferentia remaining after electroejaculation was not considered. The recovery index was calculated the following:

$$A={\text{VDW}}_{Et_{1}}-{\text{VDW}}_{Et_{0}}=\text{Increment in dry weight of seminal material after}\;t_{1}$$

$$B={\text{VDW}}_{Ct_{0}}-{\text{VDW}}_{Et_{0}}=\text{Quantity of seminal material to be recovered}$$

$$\text{RI}\;\left(\%\right)=\frac{A}{B}\times 100$$

VDW = Vasa deferentia dry weight; E = Crab electroejaculated at beginning of experiment; C = Control (i.e., without electroejaculation); *t*_0_ = At beginning of the experiment; and *t*_1_ = After corresponding time period of either 15 or 30 days.

The RIs were calculated with the formulas above using bootstrapping (replications = 1,000; “boot” function of the “boot” package; Davison & Hinkley, 1997; Canty & Ripley, 2017). Bootstrapped 95% confidence intervals (*Bca* method) were generated for the RIs. Due to bootstrapping of data, specific statistical tests were not performed and significant difference between two groups was determined based on the 95% confidence intervals. Compared RIs were considered significantly different from each other when the 95% confidence intervals did not overlap.

An ANOVA with permutation was used due to non‐normal distribution to evaluate whether the three factors, time period, temperature, and food availability, and the interactions between them affected the hepatosomatic index in initially ejaculated crabs. Contrasts with permutation were performed to detect differences in the hepatosomatic index between the initial values and each fed and food‐deprived groups with initial electroejaculation. “Aovp” and “lmp” functions of the package “lmPerm” were used (Wheeler & Torchiano, 2016). To check for no differences in crab sizes among groups, a one‐way ANOVA was performed. A linear regression was performed to test the relationship between vasa deferentia dry weight and carapace length of crabs. We performed a one‐way ANCOVA with season as a fixed factor and carapace size as a covariate to test whether seasonal variation in vasa deferentia dry weight depended on carapace length of crabs. For the regression analysis and the one‐way ANCOVA, we used crabs without electroejaculation (i.e., control) and in addition data of dry weight of vasa deferentia of seasonally collected and dissected crabs (Los Molinos Bay, January 2016–May 2017; Pretterebner, 2019) to achieve a larger sample size (*n* = 28).

All data were checked for normality using the Shapiro–Wilk test. The Bartlett test was applied to check for variance homogeneity. All statistical analyses were performed using the software R version 3.4 (R Core Team, 2017).

## 3 RESULTS

**Figure 2:**
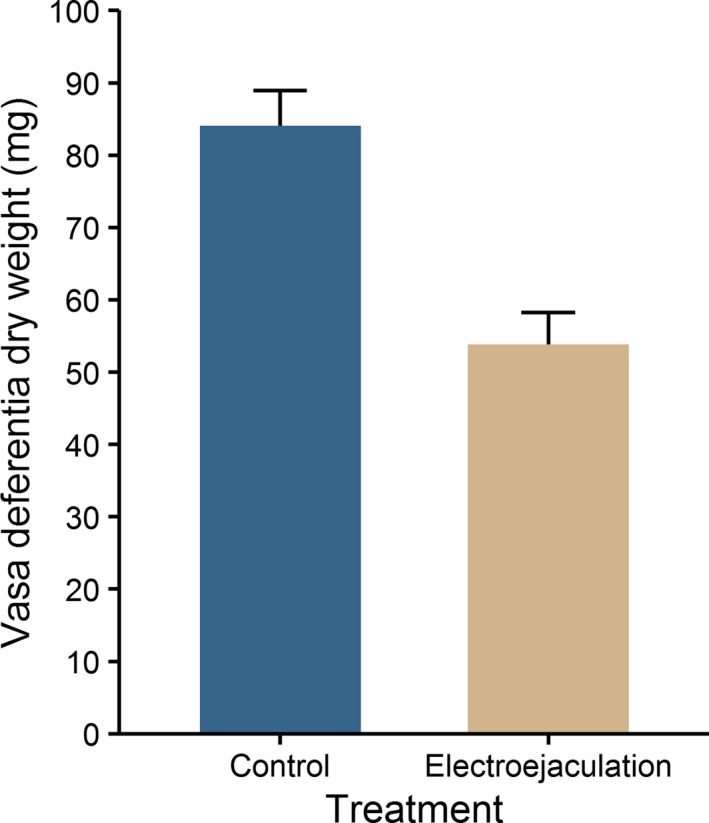
Mean + *SE* initial vasa deferentia dry weight of *Taliepus dentatus* before and after electroejaculation (*n* = 4 in each group)

Males of *T. dentatus* were similar‐sized (mean carapace length = 98.6 ± 0.6 mm), and no size difference existed among crabs of all experimental groups (one‐way ANOVA, *F*_13,__52_ = 0.22, *p* = .998). The vasa deferentia dry weight of males was not related to carapace length (equation: vasa deferentia dry weight = 34.195 + 0.778 * (carapace length); *R* ^2^ = −0.02, *F*_1,__26_ = 0.46, *p* = .502). In the one‐way ANCOVA, the covariate, carapace length, was not significant (*F*_1,__23_ = 0.83, *p* = .371) and vasa deferentia dry weight varied seasonally (*F*_3,__23_ = 7.87, *p* < .001). The results of the regression and the ANCOVA suggest that there is no need to include crab size in the calculation of the recovery index. Initial dry weight of paired vasa deferentia without electroejaculation was 84 ± 4 mg and after electroejaculation 52 ± 4 mg (Figure 2). Therefore electroejaculation reduced the initial vasa deferentia dry weight by 38%.

**Figure 3:**
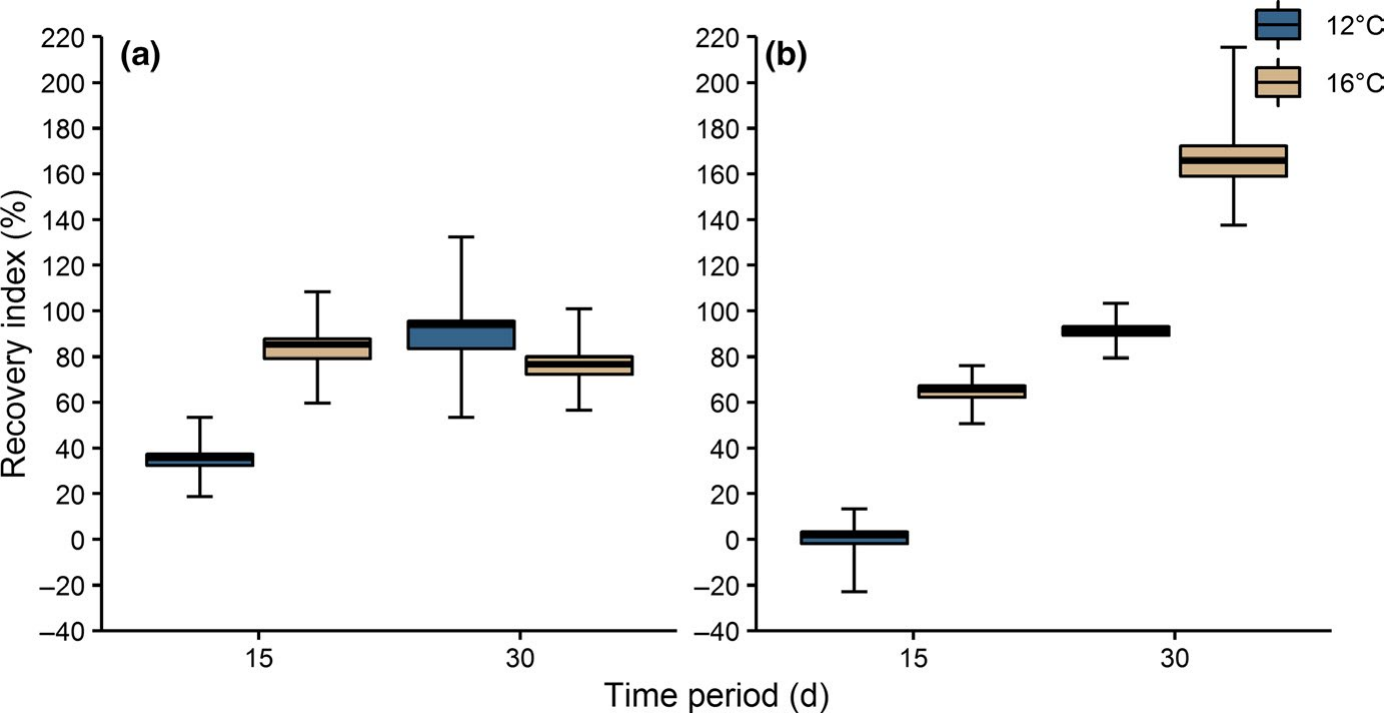
Recovery index for the corresponding time periods and maintenance water temperatures of *Taliepus dentatus* in distinct regimes of alimentation: (a) ad libitum; and (b) food deprivation. Box plot depicts mean, first and third quartiles, and 95% confidence intervals of the mean (whiskers) estimated from 1,000 bootstrap replicates. Recovery index calculated based on *n* = 4–5 in each group

After 15 days in both food regimes, the recovery index was significantly higher at 16°C than 12°C (Figure 3a,b). The recovery index at 12°C in both food regimes and at 16°C with food deprivation increased significantly between 15 and 30 days. After 30 days with food deprivation, the recovery index was significantly higher at 16 than 12°C. After 30 days, the recovery index of both, ad libitum food and food deprivation, at 12 and 16°C reached to at least 100%; thus, seminal reserves were fully recovered in all treatments. However, food‐deprived males at 16°C after 30 days overcompensated and recorded the highest mean recovery index (165%; Figure 3b).

**Figure 4:**
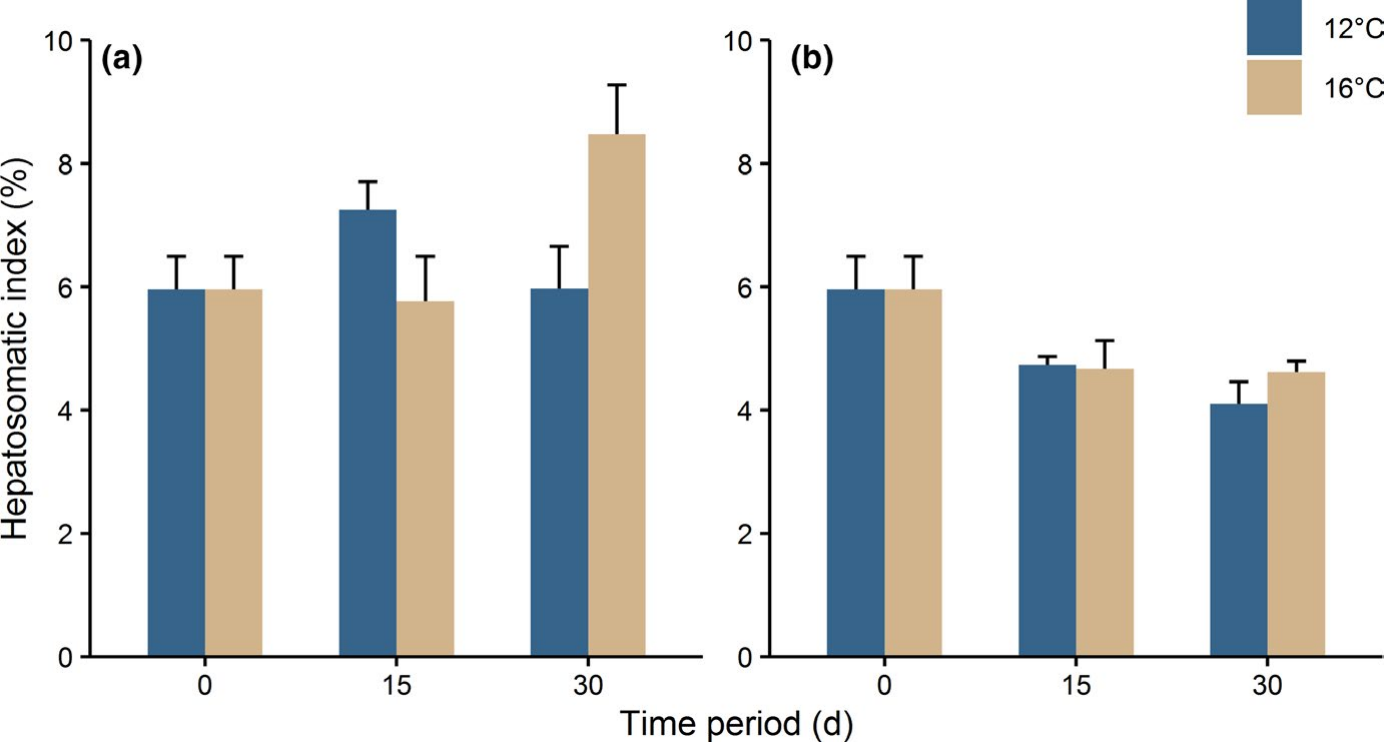
Hepatosomatic index of *Taliepus dentatus* after the corresponding recovery periods with and without electroejaculation in two distinct regimes of alimentation: (a) ad libitum; and (b) food deprivation. Values are means + *SE* (*n* = 4–5 in each group)

Mean initial hepatosomatic index for electroejaculated crabs was 6.0 ± 0.5%. The hepatosomatic index was significantly affected by food availability and the interaction of food availability and time period (Table 1). After 30 days with ad libitum food, mean hepatosomatic index was 6.0 ± 0.7% and 8.5 ± 0.8% at 12 and 16°C, respectively (Figure 4a). The hepatosomatic index was significantly increased after 30 days at 16°C (Table 2). The hepatosomatic index decreased significantly after 30 days in the food‐deprived groups at both 12°C (mean hepatosomatic index = 4.1 ± 0.4%) and 16°C (mean hepatosomatic index = 4.6 ± 0.2%; Table 2 and Figure 4b) compared with the initial values.

**Table 1:** Results of ANOVA with permutation to evaluate whether the three factors, time period, temperature, and food availability, and the interactions between them affected the hepatosomatic index in *Taliepus dentatus* including only initially ejaculated crabs

Factors	*df*	MS	No. of iterations	*p*
Time	1	0.511	103	.495
Temp.	1	0.531	68	.602
Food	1	32.332	5,000	**<.001**
Time * temp.	1	4.801	1,264	.0735
Time * food	1	18.511	5,000	**<.001**
Temp. * food	1	0.065	51	.901
Time * temp. * food	1	2.173	597	.144
Residuals	50	1.521		

Bold values indicate statistical significance at the *p* < .05 level.

**Table 2:** Results of performed contrasts with permutation to detect differences in hepatosomatic index of *Taliepus dentatus* between the initial values with electroejaculation and each fed and food‐deprived recovery groups

Time period (d)	Temperature (°C)	Food	Estimate	No. of iterations	*p*
15	12	Ad libitum	1.292	226	.309
15	16	Ad libitum	0.193	262	.278
30	12	Ad libitum	−0.014	387	.206
30	16	Ad libitum	−2.517	5,000	**.019**
15	12	Deprived	−1.223	61	.623
15	16	Deprived	1.292	51	.705
30	12	Deprived	1.857	5,000	**<.001**
30	16	Deprived	1.340	4,574	**.021**

Bold values indicate statistical significance at the *p* < .05 level.

## 4 DISCUSSION

Male crabs exhibited plasticity in recovery of seminal reserves in response to distinct environmental conditions. Especially prolonged starvation at 16°C triggered increased allocation of energy to reproduction. Contrary as expected, the recovery index at 16°C within 30 days was twice as high in starved compared with fed crabs. Environmental conditions may determine the costs and benefits of an organism's life history strategy, and consequently in the face of changes in the environmental conditions, the trade‐offs between costs and benefits can change. The reproductive effort may be altered in an environment where individuals are exposed to different levels of a threat to future reproduction in favor of plastic strategies of energy allocation to reproduction. Similarly, prolonged food deprivation at 16°C may represent extremely suboptimal conditions for *T. dentatus*, triggering a concentration of the energy investment to the current effort in reproduction. Male crabs likely adapted a plastic strategy similar as proposed by the terminal investment hypothesis. The terminal investment hypothesis refers to the idea that individuals facing a decreased expectation of future reproduction, such as due to threat of mortality, should elicit increased investment in current reproduction (Clutton‐Brock, 1984; Hirshfield & Tinkle, 1975; Williams, 1966). Terminally investing species have been demonstrated in diverse taxa of vertebrates and invertebrates (reviewed by Duffield, Bowers, Sakaluk, & Sadd, 2017). For example, food‐deprived males of the yellow mealworm beetle *Tenebrio molitor* invest in their sexual attractiveness, however, at the expense of their immune response and survival (Krams et al., 2015). In *T. dentatus,* the combination of temperature and prolonged starvation likely served as a cue to induce increased energy allocation to reproduction. Under the combined effects of high temperature and food deprivation, males should be able to fertilize one additional female with the extra amount of seminal material, as they transfer approximately 37% of their seminal material stock during one mating to females (Pretterebner, 2019) which corresponds to the amount of seminal material extruded during electroejaculation in this study.

Complete replenishment of seminal reserves occurred at the latest within 30 days in all treatments in *T. dentatus*. Seminal recovery period seems to be relatively fast in *T. dentatus* compared with the southern king crab *L. santolla* which requires more than 30 days for recovery at 12°C (Pretterebner et al., 2019). Matings in *T. dentatus* occur permanently and during nearly all months of the year in the laboratory, indicating a continuous reproductive pattern (Pretterebner, 2019). Fast recovery may be necessary to perform extensive reproductive activities. This species has a determinate growth pattern (Pretterebner, 2019), which may also allow males to allocate a great amount of energy to reproduction and facilitate recovery. This suggests that the seminal recovery rate may be closely linked to the specific mating strategy of this species.

Variation in the recovery rate was dependent on temperature and availability of food. Temperature affected the period of seminal recovery, which was faster in seawater of 16°C (15 days) than that of 12°C (30 days). However, the recovery index was higher at both temperatures in fed than food‐restricted crabs after 15 days of experimentation. Full recovery was first reached in fed crabs after 15 days at 16°C, which represented the treatment with the fastest recovery recorded. This suggests that favorable feeding conditions during summer months may accelerate seminal recovery. Faster refilling of vasa deferentia after mating may enable males to mate more frequently and/or deliver larger ejaculates to females, thereby increasing their reproductive output. In comparison, in the king crab *L. santolla* originating from its northern distributional limit, the effect of temperature on seminal recovery is the contrary, as it is twice as fast in seawater of 9°C than 12°C. The latter may be close to the king crab's temperature threshold. Another example is the red claw crayfish *Cherax quadricarinatus* in which sperm production is higher at 27 and 29°C than at 23 and 31°C after 150 days (Bugnot & López Greco, 2009). This indicates that the specific effect of temperature on seminal recovery varies depending on the species and its origin. Probably, similar to the general functioning of the organism, an optimal temperature range may also exist for the production of seminal material in which recovery occurs more efficiently. The intensity of the effect of varying temperature on the recovery rate may be species‐specific and associated with the habitat and geographical distribution of a species (Table 3).

**Table 3:** Summary of sperm and seminal recovery in decapod species

Species (Family)	Species distribution	Temperature (°C)	Recovery period (days)	Reproductive unit	Depletion method	Reference
*Taliepus dentatus* (Epialtidae)	Subtropical/temperate	12	30	RI (VDW)	Electroejaculation	This study
		16	15	RI (VDW)	Electroejaculation	
*Callinectes sapidus* (Portunidae)	Subtropical	a	9–20	VDW	2 matings	Kendall et al. (2001)
		a	9–20	Sperm number	2 matings	
*Penaeus setiferus* (Penaeidae)	Tropical	25	8	Sperm number	Electroejaculation	Pascual et al. (1998)
		30	~6	Sperm number	Electroejaculation	
		33	6	Sperm number	Electroejaculation	
*Penaeus brasiliensis* (Penaeidae)	Tropical	27	16	Spermatophores (macroscopic evaluation)	Manual extrusion	Braga, Lopes, Poersch, and Wasielesky (2014)
*Lithodes santolla* (Lithodidae)	Temperate/cold‐temperate	9	>30	RI (VDW)	Electroejaculation	Pretterebner et al. (2019)
		12	>30	RI (VDW)	Electroejaculation	
		9	>30	Sperm area	Electroejaculation	
		12	>30	Sperm area	Electroejaculation	
*Paralithodes brevipes* (Lithodidae)	Temperate/cold‐temperate	a	>28	Sperm number	Various matings	Sato et al. (2006)
*Hapalogaster dentata* (Lithodidae)	Temperate/cold‐temperate	a	>20	Sperm number	Various matings	Sato and Goshima (2006)

^a^ Indicates studies with unstable temperature conditions. RI and VDW refer to recovery index and vasa deferentia dry weight, respectively.

In food‐deprived crabs at 12°C after 15 days, no recovery was detected, displayed in the recovery index close to zero percent. Another effect of availability of food was also reflected in the decreased hepatosomatic index in the starvation treatment compared with ad libitum. In crabs with ad libitum alimentation, the hepatosomatic index was relatively constant. At 16°C between 15 and 30 days, crabs even started to accumulate reserves in the hepatopancreas while no further seminal recovery occurred during this period. At 16°C after 30 days, no further energy was allocated to seminal recovery and the surplus of energy may be manifested in an increased hepatosomatic index. The blue crabs *Callinectes danae* (Zara, Toyama, Caetano, & López Greco, 2012) and *C. ornatus* (Aparecida do Nascimento & Zara, 2013) and shrimp species (Castille & Lawrence, 1989) are examples which use part of their reserves stored in the hepatopancreas to develop the male reproductive system. Especially in female crabs, the mobilization of energy from the hepatopancreas during gonadal development has been documented (Castiglioni, Oliveira, & Bond‐Buckup, 2006; Chu, 1999; Kyomo, 1988; López Greco & Rodríguez, 1999). In contrast, the effect of starvation in males of *T. dentatus* was manifested in a decreased hepatosomatic index already after 15 days. Similarly, as a consequence of starvation, a decline in the hepatosomatic index has been observed in *L. santolla* (Comoglio, Goldsmit, & Amin, 2008) and in shrimps (Cuzon et al., 1980; Sánchez‐Paz et al., 2007).

Interspecific comparison of the recovery periods is difficult among decapods because the type of information reported related to the male reproductive organ, method applied to deplete seminal material stocks and experimental temperatures are inconsistent (Table 3). Comparing the species studied so far, a general trend in the duration of sperm or seminal recovery has been observed depending on the distribution of the species: A faster recovery rate may occur in tropical/subtropical species, in contrast, to slower replenishment in cold‐temperate species (Table 3). Males of *T. dentatus* produced abundant spermatophores and seminal fluids, and rapid recovery likely involved high costs in terms of energy mobilized from the hepatopancreas. In particular, males exhibited the ability of a flexible energy allocation in the context of environmental stress, which may give this species an adaptive advantage and explain its ecological success. This strategy seems to be very successful because it allows for a high intensity of reproduction, which might have contributed to *T. dentatus* being one of the most abundant species in subtidal environments.

## 5 CONCLUSIONS

The fast seminal recovery in males of *T. dentatus* may be a strategy to allow extensive reproductive activities year‐round. The kelp crab *T. dentatus* is distributed over a large range of latitudes, along which it is exposed to environmental fluctuations, which may result in different seminal recovery rates. Overcompensation of seminal recovery in male crabs in the face of food deprivation provides an example of an adaptive response under an environmental variation scenario.

## CONFLICT OF INTEREST

None declared.

## AUTHOR CONTRIBUTIONS

KP conceived the study, collected and analyzed data, and drafted the manuscript. LMP conceived the study, and analyzed and interpreted data. All authors revised the manuscript.

## Data Availability

Data have been archived in the Dryad repository at https://doi.org/10.5061/dryad.kprr4xh1r.
